# Innovation During Disruption: Supporting What Matters Most to Older Adults Through Person-Environment Fit

**DOI:** 10.1093/geroni/igab046.1125

**Published:** 2021-12-17

**Authors:** Sarah Szanton

**Affiliations:** Johns Hopkins University, Baltimore, Maryland, United States

## Abstract

The fragile and improvised systems of care for older adults have been decimated by isolation and fragmented care during the pandemic. However, innovations are increasingly being offered to older adults to improve the fit between them and their environment. This includes fit within the home, the social environment, the policy environment, and with clinicians. Advancing these “fits” requires evidence-based solutions like CAPABLE, a 4 month self-efficacy and function program that provides an occupational therapist, nurse and handyworker to assess and address older adults’ functional goals. The older adult identifies what matters most and experiences a tailored program that taps into their purpose in life and supports engaging in meaningful activities. Starting in research sites, CAPABLE is now offered in 34 sites in 17 States and expanding through policy and insurers. Such efforts to leverage the strength of older adults and their families, builds capacity to evolve our communities of care.

